# Faecal microbiota transplantation reduces amounts of antibiotic resistance genes in patients with multidrug-resistant organisms

**DOI:** 10.1186/s13756-022-01064-4

**Published:** 2022-01-29

**Authors:** JongHoon Hyun, Sang Kil Lee, Jae Hee Cheon, Dong Eun Yong, Hong Koh, Yun Koo Kang, Moo Hyun Kim, Yujin Sohn, Yunsuk Cho, Yae Jee Baek, Jung Ho Kim, Jin Young Ahn, Su Jin Jeong, Joon Sup Yeom, Jun Yong Choi

**Affiliations:** 1 Division of Infectious Disease, Department of Internal Medicine, Gangnam Severance Hospital, Yonsei University College of Medicine, Seoul, South Korea; 2 Division of Gastroenterology, Department of Internal Medicine, Yonsei University College of Medicine, Seoul, South Korea; 3 Division of Laboratory Medicine and Research Institute of Bacterial Resistance, Department of Internal Medicine, Severance Hospital, Yonsei University College of Medicine, Seoul, South Korea; 4 Division of Gastroenterology, Hepatology, and Nutrition, Department of Pediatrics, Severance Children’s Hospital, Severance Pediatric Liver Disease Research Group, Yonsei University College of Medicine, Seoul, South Korea; 5 Division of Gastroenterology, Hepatology, and Nutrition, Department of Pediatrics, Yonsei University Wonju College of Medicine, Wonju, South Korea; 6 Division of Infectious Diseases, Department of Internal Medicine, Yonsei University College of Medicine, Yonsei University Health System, 50-1, Yonsei-ro, Seodaemun-gu, Seoul, 03722 South Korea

**Keywords:** Faecal microbiota transplantation, Multidrug-resistant organism, Vancomycin-resistant enterococci, Carbapenemase-producing Enterobacteriaceae, Microbiome

## Abstract

**Background:**

Multidrug-resistant organisms (MDROs) such as vancomycin-resistant enterococci (VRE) and carbapenemase-producing Enterobacteriaceae (CPE) are associated with prolonged hospitalisation, increased medical costs, and severe infections. Faecal microbiota transplantation (FMT) has emerged as an important strategy for decolonisation. This study aimed to evaluate the genetic response of MDROs to FMT.

**Methods:**

A single-centre prospective study was conducted on patients infected with VRE, CPE, or VRE/CPE who underwent FMT between May 2018 and April 2019. Genetic response was assessed as the change in the expression of the resistance genes *VanA*, *bla*_KPC_, *bla*_NDM_, and *bla*_OXA_ on days 1, 7, 14, and 28 by real-time reverse-transcription polymerase chain reaction.

**Results:**

Twenty-nine patients received FMT, of which 26 (59.3%) were infected with VRE, 5 (11.1%) with CPE, and 8 (29.6%) with VRE/CPE. The mean duration of MDRO carriage before FMT was 71 days. Seventeen patients (63.0%) used antibiotics within a week of FMT. In a culture-dependent method, the expression of *VanA* and overall genes significantly decreased (*p* = 0.011 and *p* = 0.003 respectively). In a culture-independent method, *VanA*, *bla*_NDM_, and overall gene expression significantly decreased over time after FMT (*p* = 0.047, *p* = 0.048, *p* = 0.002, respectively). Similar results were confirmed following comparison between each time point in both the culture-dependent and -independent methods. Regression analysis did not reveal important factors underlying the genetic response after FMT. No adverse events were observed.

**Conclusion:**

FMT in patients infected with MDROs downregulates the expression of resistance genes, especially *VanA*, and facilitates MDRO decolonisation.

## Background

The increase in the rate of antibiotic resistance is one of the most important public health concerns worldwide [1–3]. The human gut is a complex microbial ecosystem of symbiotic gastrointestinal (GI) microorganisms [4]. Dysbiosis owing to antibiotics can disrupt the colonisation of non-commensal pathogens and encourage the colonisation of multidrug-resistant microorganisms (MDROs) [4, 5]. The gut microbiome is not only a potential reservoir for MDROs such as vancomycin-resistant enterococci (VRE) and carbapenemase-producing Enterobacteriaceae (CPE) but also a hub of antibiotic resistance genes such as *VanA*, *bla*_KPC_, *bla*_OXA_, and *bla*_NDM_, collectively known as the gut resistome [4, 6]. The gut resistome provides a suitable environment for the horizontal transfer of antimicrobial resistance genes through conjugation, natural transformation, and transduction [7, 8]. The transfer of antimicrobial resistance genes within commensals can create new antimicrobial-resistant pathogens and pose a challenge for the treatment of patients with severe MDRO infection. Therefore, it is imperative to develop a novel strategy that addresses the resistance environment with various antibiotic resistance genes. One of the ways to deal with the gut resistome comprising clinically important resistance genes is to reconstruct a new environment through faecal microbiota transplantation (FMT).

FMT is a way to restore the human gut microbiome by transferring the microbiota from healthy donors. It is conducted by the administration of refined faeces obtained from screened healthy donors into the colon or the upper small intestine of patients via colonoscopy, enema, or capsules [9, 10]. Its safety and effectiveness against recurrent *Clostridium difficile* infection (CDI) in the GI tract is well established [11, 12]. A recent study showed that FMT is effective in reducing antibiotic resistance genes in patients with recurrent CDI and modifying the expression of resistance genes [11–13]. Numerous studies have shown the effectiveness of FMT in decolonisation of MDROs [10, 14, 15]. FMT is thought to restore the natural microbiota by replacing MDROs with healthy bacteria, leading to the generation of diverse compositions of the human gut microbiome. Although FMT can reduce the expression of resistance genes in patients infected with CDI, its effect on MDROs has not been investigated. In the present study, we investigated whether FMT significantly reduces the expression of antibiotic resistance genes in patients colonized with MDROs.

## Materials and methods

### Subjects and study design

This was a prospective, single-centre cohort study performed between May 2018 and April 2019 at Severance Hospital in Seoul, South Korea. Patients older than 6 months colonized with CPE, VRE, or CPE and VRE in the GI tract were enrolled in this study. CPE or VRE colonisation was determined by at least one positive result of rectal swab culture a week prior to FMT. Patients carrying MDROs in a location other than the GI tract or who had immunosuppression, food allergy, or high risk associated with study participation were excluded. Patients who used antibiotics were allowed to continue to do so at the time of FMT, and there were no restrictions on the use of antibiotics after FMT. The sample size of this study was determined using the GPower 3.1 Software. Power analysis indicated that a total of 25 participants (number of measurements = 5) were needed for effect size (0.25) when α = 0.05 for a power of 0.8. Therefore, a total of 27 patients were enrolled taking dropout rate into consideration.

The primary endpoint of the study was the complete elimination of antibiotic resistance genes 1 month after FMT. The secondary endpoint included decreased expression of resistance genes. All faecal samples, regardless of MDRO subtype, were tested using real-time reverse-transcription polymerase chain reaction (PCR) for the quantification of antibiotic resistance genes such as *VanA*, *bla*_KPC_, *bla*_OXA_, and *bla*_NDM_. Genetic response was defined as a decrease in the number of genes.

### Faecal microbiota transplantation

Faecal material was obtained from healthy, unrelated donors. All volunteers were screened based on their history and clinical examination for antibiotic use within 3 months, GI symptoms, and any risk of infectious disease. Donors were excluded if they had taken any antibiotics in the past 3 months. Donors were tested for hepatitis (A, B, and C), human immunodeficiency virus, syphilis, bacteria (stool culture), rotavirus/norovirus/adenovirus (stool PCR), *C. difficile* toxin, parasites and their eggs (rectal exam), and VRE and CPE (stool culture). Stool samples were donated, and 100 g samples were mixed with 200 mL of sterile normal saline and stored as concentrated glycerol stocks at − 70 °C.

FMT was performed using a preparation of the frozen faecal solution via colonoscopy, duodenoscopy, a percutaneous jejunostomy tube, or an gastric capsule. The FMT delivery modality was selected based on the clinicians’ assessment as per the patient’s clinical condition. Patients under colonoscopy took 4 L of bowel preparations 1 day prior to FMT. Only patients who were less likely to aspirate were treated with capsules, which were taken after fasting for 2 h following breakfast for 2 consecutive days. After taking capsules with cranberry juice, patients were advised not to ingest any food and to sit in Fowler’s position for at least 2 h.

### Real-time reverse-transcription PCR for detection of antibiotic resistance genes

Faecal samples of subjects were obtained before FMT and 1, 7, 14, and 28 days after FMT and stored at − 80 °C until used for DNA extraction. The efficacy of FMT was assessed by real-time PCR to detect expression of antibiotic resistance genes encoding VanA and carbapenemase. The DNA was extracted from faecal samples using the FastDNA® SPIN Kit for Soli (MP Biomedicals, Solon, OH, USA), which is well-suited for use with faecal samples, as per the manufacturer’ s instructions. Based on the available literature data, PCR was performed using the primers VanAF 5ʹ-ATCAACCATGTTGATGTAGC-3ʹ for *VanA*, KPC-rtF 5ʹ-CAGCTCATTCAAGGGCTTTC-3ʹ for *bla*_KPC_, Oxa-rtF 5ʹ-AGGCACGTATGAGCAAGATG-3ʹ for *bla*_OXA_, and Ndm-rtF 5ʹ-GATTGCGACTTATGCCAATG-3ʹ for *bla*_NDM_. PCR was performed using 2 × SYBR Green Master Mix (Applied Biosystems) on a 7300 Real-Time PCR system (Applied Biosystems). Amplifications were carried out under the following conditions: 95 °C for 30 s; 40 cycles of 95 °C for 30 s, 60 °C for 34 s, and 95 °C for 15 s, 60 °C for 60 s, and 95 °C for 15 s. Amplification was verified by running the products on a 1% agarose gel. Standard curves were generated using the reference quantities of the cloned resistance genes.

The analysis was performed in two ways. One was culture-dependent PCR, which detects resistance genes based on the resistance the strains demonstrate in cultures. The other is culture-independent PCR, which quantifies resistance genes in polymicrobial samples regardless of their phenotypes. In the first case, for example, *VanA* gene quantified using PCR confirmed the presence of VRE. In the latter case, all resistance genes such as *VanA*, *bla*_KPC_, *bla*_OXA_, and *bla*_NDM_ were quantified regardless of the MDRO type.

### Data collection

Clinical and laboratory data at each follow-up point were collected as follows: age, sex, body mass index, type of MDRO carriage, duration of carriage before FMT, whether antibiotic treatment was used either before or after FMT, laboratory findings such as white blood cells, haemoglobin, platelets, blood urine nitrogen, creatinine, aspartate transaminase, alanine transaminase, total cholesterol, low-density lipoprotein, albumin, fasting glucose, and C-reactive protein. All DNA values were log_10_ transformed before analysis.

### Statistical analysis

All variables are presented as mean ± standard deviation, unless otherwise indicated. Comparisons were performed using Mann–Whitney U test, χ^2^ analysis, or Fisher’s exact test, as appropriate. A linear mixed model was used to confirm significant decrease in gene expression after FMT. The number of genes at each point was compared using the Wilcoxon signed-rank test. Statistical significance was set at *p* < 0.05. All statistical analyses were conducted using the Statistical Package for the Social Sciences version 25.0 (IBM Corporation, Armonk, NY, USA).

## Results

### Patient characteristics

A total of 27 patients who tested positive for MDRO were prospectively enrolled and underwent FMT. The gut colonising MDROs included VRE and CPE. The most common enterococci were *Enterococcus faecium* (n = 26), and most of the Enterobacteriaceae were *Klebsiella pneumoniae* (n = 11).

The clinical characteristics of the patients are summarised in Table 1. The median age of the participants was 51.23 years (interquartile range [IQR] = 27.24), and 16 patients were male (59.3%). Fifteen patients (55.5%) were tested positive for VRE, 1 (3.7%) was tested positive for CPE, and 11 (40.7%) for VRE and CPE. Antibiotic resistance genes included *VanA* (n = 26), *bla*_KPC_ (n = 11), and *bla*_OXA_ (n = 1). The duration of MDRO colonisation before FMT was 71 days. Sixteen patients (59.3%) were treated with antibiotics before FMT, and 17 patients (62.9%) were treated with antibiotics after FMT.

**Table 1:** Baseline characteristics of study participants (n = 27)

Characteristics	
Age, years	51.23 ± 27.24
Sex, male	16/27 (59.3%)
BMI, kg/m^2^	20.85 ± 4.84
MDRO carriage	
VRE	15 (55.5%)
CPE	1 (3.7%)
VRE/CPE	11 (40.7%)
Antibiotic resistance gene	
*VanA*	26 (68.4%)
*bla*_KPC_	11 (28.9%)
*bla*_OXA_	1 (2.6%)
*bla*_NDM_	0
Duration of carriage before FMT, days	71.00 ± 88.00
Antibiotics use before FMT within 1 week	16 (59.3%)
Antibiotics use after FMT within 1 week	17 (62.9%)
Laboratory test at FMT	
WBC count, 10^3^/μL	6.88 ± 2.53
Haemoglobin, g/dL	10.68 ± 1.48
Platelet count, 10^3^/μL	293.32 ± 138.38
BUN, mg/dL	15.07 ± 12.78
Creatinine, mg/dL	15.07 ± 12.78
AST, IU/L	37.20 ± 31.51
ALT, IU/L	23.36 ± 15.59
Total cholesterol, mg/dL	161.12 ± 54.55
LDL cholesterol, mg/dL	94.32 ± 40.48
Albumin, mg/dL	3.38 ± 0.51
Fasting glucose, mg/dL	108.24 ± 39.94
CRP, mg/L	13.61 ± 13.71

Values are expressed as number of patients (%), if not otherwise described

FMT, faecal microbiota transplantation; BMI, body mass index; MDRO, multidrug-resistant organism; VRE, vancomycin-resistant enterococci; CPE, carbapenemase-producing Enterobacteriaceae; WBC, white blood cell; BUN, blood urine nitrogen; AST, aspartate transaminase; ALT, alanine transaminase; LDL, low-density lipoprotein; CRP, C-reactive protein

### Genetic responses

Microbiological follow-up data were available for all patients with FMT at 1, 7, 14, and 28 days. Real-time reverse-transcription PCR was performed to detect antibiotic resistance genes in all stool samples. In addition to the previously identified resistance genes, a test was also conducted on the remaining resistance genes using the culture-independent method.

The expression of *VanA* significantly decreased from 9.28 log_10_ to 6.82 log_10_ copies/mL on day 28 of FMT (*p* = 0.011), and that of *bla*_KPC_ decreased from 7.41 log_10_ to 5.14 log_10_ copies/mL, but no significant difference was observed (*p* = 0.126). The expression of all genes, including *VanA*, and *bla*_KPC_, was significantly downregulated from 8.57 log_10_ to 6.34 log_10_ copies/mL over time after FMT (*p* = 0.003) (Table 2). We measured the expression levels of all antibiotic resistance genes in each sample and found that the expression levels of *VanA* and *bla*_NDM_ decreased from 9.41 log_10_ to 7.56 log_10_ and 5.86 log_10_ to 2.80 log_10_ copies/mL, respectively (*p* = 0.047 and *p* = 0.048). However, no significant decrease was observed in the expression levels of *bla*_KPC_ and *bla*_OXA_, as evident from a decrease from 8.00 log_10_ to 6.13 log_10_ and 2.06 log_10_ to 1.72 log_10_ copies/mL, respectively (*p* = 0.063 and *p* = 0.774). The levels of all genes, including *VanA*, *bla*_KPC_, *bla*_OXA_, and *bla*_NDM_, significantly decreased over time from 9.64 log_10_ to 6.04 log_10_ copies/mL (*p* = 0.002) (Table 3).

**Table 2:** Quantification of resistance gene expression after FMT in a culture-dependent method

	Pre	Day 1	Day 7	Day 14	Day 28	p-value
*VanA* gene, log_10_ copies/mL	9.28 ± 2.05	8.45 ± 1.87	8.82 ± 2.39	7.60 ± 2.84	6.82 ± 3.53	0.011
*bla*_KPC_ gene, log_10_ copies/mL	7.41 ± 3.29	7.30 ± 3.31	6.11 ± 3.58	6.47 ± 4.14	5.14 ± 4.60	0.126
Overall gene	8.57 ± 2.76	8.10 ± 2.36	8.01 ± 3.05	7.15 ± 3.24	6.34 ± 3.75	0.003

FMT, faecal microbiota transplantation

**Table 3:** Quantification of resistance gene expression after FMT in a culture-independent method

	Pre	Day 1	Day 7	Day 14	Day 28	p-value
*VanA* gene, log_10_ copies/mL	9.41 ± 2.34	8.59 ± 1.96	8.91 ± 2.70	7.82 ± 3.06	7.56 ± 3.02	0.047
*bla*_KPC_ gene, log_10_ copies/mL	8.00 ± 2.62	6.83 ± 3.20	5.48 ± 2.92	6.01 ± 3.63	6.13 ± 4.18	0.063
*bla*_OXA_ gene, log_10_ copies/mL	2.06 ± 1.83	1.83 ± 1.40	2.11 ± 1.87	2.56 ± 2.34	1.72 ± 0.82	0.774
*bla*_NDM_ gene, log_10_ copies/mL	5.86 ± 3.63	4.04 ± 2.36	3.22 ± 0.81	3.24 ± 0.93	2.80 ± 0.79	0.048
Overall gene	9.64 ± 2.60	8.56 ± 2.53	8.52 ± 3.42	7.06 ± 3.90	6.04 ± 4.27	0.002

FMT, faecal microbiota transplantation

**Fig. 1:**
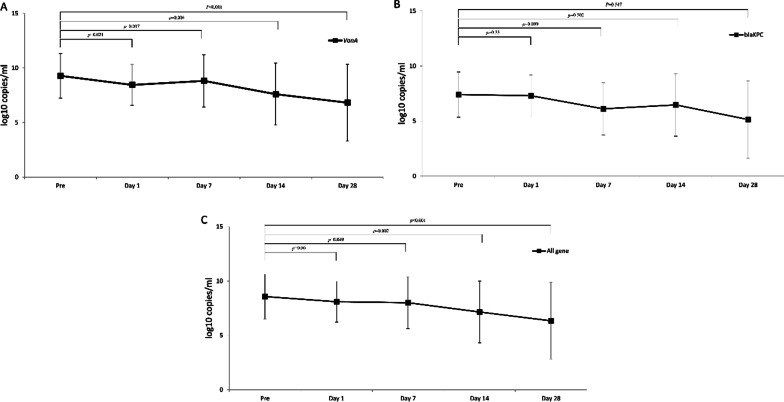
Genetic response after FMT in culture-dependent method. **A** Changes in amount of *VanA* gene. **B** Changes in amount of *bla*_KPC_ gene. **C** Changes in amount of all resistance genes including *VanA*, *bla*_KPc_, *bla*_OXA_, and *bla*_NDM_

**Fig. 2:**
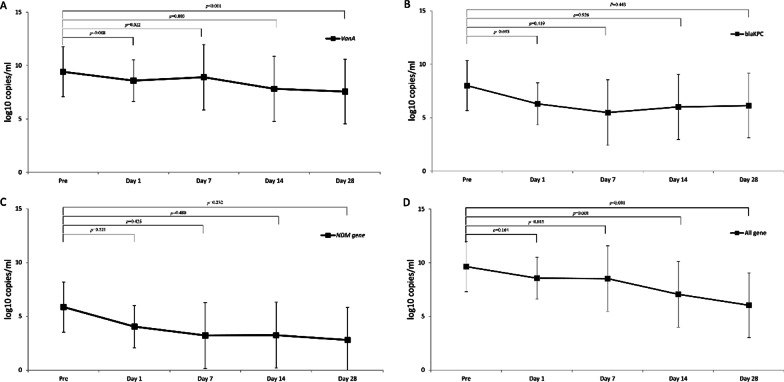
Genetic response after FMT in culture-independent method. **A** Changes in amount of *VanA* gene. **B** Changes in amount of *bla*_KPC_ gene. **C** Changes in amount of *bla*_NDM_ gene. **D** Changes in amount of all resistance genes including *VanA*, *bla*_KPc_, *bla*_OXA_, and *bla*_NDM_

We analysed the decrease in the expression of genes at each time point. *VanA* gene expression level significantly decreased between pretreatment and day 1, pretreatment and day 7, pretreatment and day 14, and pretreatment and day 28 (*p* = 0.021, 0.017, 0.004, < 0.001, respectively). No significant reduction in the expression of *bla*_KPC_ was observed between these time points (*p* = 0.33, 0.099, 0.502, 0.547, respectively). The levels of all genes, including *VanA* and *bla*_KPC_, significantly decreased between pretreatment and day 7, pretreatment and day 14, and pretreatment and day 28 but not between pretreatment and day 1. (*p* = 0.049, 0.003, < 0.001, 0.06, respectively) (Fig. 1). Similar results were obtained when we tested expression of all antibiotic resistance genes in each sample. *VanA* gene expression significantly decreased at all time points (p = 0.008, 0.022, 0.003, < 0.001), but no statistically significant decrease was observed in *bla*_KP_, *bla*_OXA_, and *bla*_NDM_ gene expression levels. The number of genes expressed, including *VanA*, *bla*_KPc_, *bla*_OXA_, and *bla*_NDM_, significantly decreased between pretreatment and day 7, pretreatment and day 14, and pretreatment and day 28 but not between pretreatment and day 1. (*p* = 0.015, 0.008, < 0.001, 0.164, respectively) (Fig. 2).

### Factors associated with genetic responses

The results of univariate regression analyses to test the downregulation in the expression of antibiotic resistance genes are shown in Table 4. MDRO carriage (CPE: hazard ratio [HR] = 0.417, 95% confidence interval [CI] = 0.46–1.188, *p* = 0.0.102; combined VRE and CPE: HR = 0.63, 95% CI = 0.182–2.179, *p* = 0.465), antibiotic resistance gene type (*bla*_KPc_: HR = 0.620, 95% CI = 0.078–4.947, *p* = 0.652; *bla*_OXA_ HR = 0.831, 95% CI = 0.09–6.959, *p* = 0.864), duration of MDRO carriage before FMT (HR = 0.99, 95% CI = 0.994–1.004, p = 0.670), antibiotics used before FMT within 1 week (HR = 0.609, 95% CI = 0.249–1.489, *p* = 0.277), and antibiotics used after FMT (HR = 1.312, 95% CI = 0.472–3.651, *p* = 0.603) were not related to the decrease in gene expression. We did not find a significant independent predictor associated with decrease in antibiotic resistance gene expression after FMT.

**Table 4:** Significant predictors associated with genetic response after FMT

	Univariate analysis		
	Unadjusted hazard ratio	95% CI	*p*-value
Age	1.008	0.92–1.024	0.597
Sex	0.499	0.190–1.307	0.149
MDRO carriage			
VRE	1.000	Reference	0.261
CPE	0.417	0.46–1.188	0.102
VRE/CPE	0.630	0.182–2.179	0.465
Antibiotic resistance gene			
*VanA*	1.000	Reference	0.773
*bla*_KPC_	0.620	0.078–4.947	0.652
*bla*_OXA_	0.831	0.099–6.959	0.864
Duration of carriage before FMT	0.999	0.994–1.004	0.670
Antibiotics used before FMT within 1 week	0.609	0.249–1.489	0.277
Antibiotics used after FMT within 1 week	1.312	0.472–3.651	0.603

FMT, faecal microbiota transplantation; MDRO, multidrug-resistant organism; VRE, vancomycin-resistant enterococci; CPE, carbapenemase-producing Enterobacteriaceae

## Discussion

FMT is an emerging therapeutic strategy against MDRO decolonisation. The effectiveness of FMT in treating recurrent *C. difficile* infection is relatively well-established, with a resolution rate of up to 90% [11, 16, 17]. In patients colonized with MDROs, the spontaneous resolution or clearance rate is 9%–50% and may need a long time [18–20]. Multiple groups have studied the decolonisation of MDRO after FMT, which had an efficacy of approximately 50–87.5% [14, 15, 21]. FMT restores the gut microbial diversity, interacts with commensal bacteria, and increases resistance to colonisation [22–24]. In our previous study, we observed significant effects of FMT on the decolonisation of MDROs and induction of microbiota richness and biodiversity [15].

The human gut serves as an ideal reservoir of antibiotic resistance genes. Under antibiotic pressure, MDROs predominantly grow in the GI tract and express and disseminate their resistance genes via horizontal gene transfer through conjugation, transformation, or transduction [6–8, 25]. This phenomenon occurs easily because the lower GI tract exhibits a high cell density and cell-to-cell contact, high biodiversity with over 500 species, and biofilm-protective cells [7, 8]. A study on gram-negative clinical cultures showed increased susceptibility of post-FMT faecal samples to nitrofurantoin, trimethoprim-sulfamethoxazole, and aminoglycoside [12]. The expression and diversity of antibiotic resistance genes were higher in patients with recurrent CDI than in controls and significantly decreased following FMT-mediated improvement in the disease status [11]. These results provide evidence that the decrease in the expression of antibiotic resistance genes may be associated with a clinical response.

In the present study, we assessed the effect of FMT on antibiotic resistance gene clearance in patients colonized with MDROs. The patients were colonised with heterogeneous strains such as VRE, CPE, or CPE/VRE. First, we detected the expression of resistance genes from faecal cultures by PCR based on the sequences of known genes. After FMT, the expression of *VanA* significantly decreased; although the expression of *bla*_KPc_ also decreased, the difference was not significant, and *bla*_OXA_ and *bla*_NDM_ gene expression was difficult to statistically analyse owing to the small sample size. It decreased in terms of the overall study period and decreased in terms of each section. This observation is consistent with the fact that FMT more quickly and easily affects the decolonisation of VRE [15]. These results suggest that FMT is an effective way to reduce antibiotic resistance gene expression in patients colonized with MDROs.

Although we performed culture-dependent PCR to detect the expression of antibiotic resistance genes, there are still many uncultured isolates constituting the gut microbiota; thus, the traditional techniques may not completely analyse the antibiotic resistome [4, 6, 25]. To overcome this problem, we detected and quantified the levels of total community antimicrobial resistance genes. In culture-independent PCR, we quantified resistance gene expression in polymicrobial samples, regardless of their phenotypes. In VRE stool samples, *bla*_KPc,_ *bla*_OXA_, and *bla*_NDM_ genes were detected in addition to *VanA* gene; similar results were recorded for all other samples. We compared the effects after FMT and found that *VanA* and *bla*_NDM_ gene expression levels significantly decreased; *bla*_KPc_ gene expression level also decreased, but the difference was not significant. The expression of *bla*_OXA_ gene was not downregulated. These results were similar to those of the culture-dependent method. Thus, these findings suggest that the gut resistome in patients colonized with MDROs is affected by FMT.

The exposure of the human microbiota to antibiotics may selectively increase the abundance of resistant organisms owing to their growth advantage and rapid proliferation [6]. This exacerbates the dissemination of resistance genes by horizontal gene transfer in the presence of a basal gene level, leading to a vicious cycle and an increase in the number of resistant organisms [6, 26, 27]. Among patients with recurrent CDI, the number of antibiotic resistance genes significantly reduced only in those who had a clinical response after FMT. This effect was attributed to the changes in the microbial composition of the gut microbiome [11]. In our study, *VanA*, *bla*_KPc_, *bla*_OXA_, and *bla*_NDM_ gene expression decreased after FMT, but no significant decrease in *bla*_KPc_ or *bla*_OXA_ gene expression was observed. This is because several species (e.g., *Barnesiella* spp.) in the microbiota directly inhibit MDROs, especially VRE [28, 29]. In this respect, it is important to evaluate not only the clinical response but also the genetic response to understand the effect of FMT.

Our study has several limitations. First, the sample size was small. In addition, we did not compare the control group with patients colonized with MDROs but who were not treated with FMT. We also did not determine the clinical impact of resistance gene clearance after FMT. Further studies are warranted to determine the relationship between antibiotic resistance gene clearance and clinical outcomes.

## Conclusion

This study shows that FMT has many beneficial effects not only through MDRO decolonisation and restoration of composition but also through the reduction in the number and expression of antibiotic resistance genes in the gut microbiome. FMT is especially effective in *VanA* and *bla*_NDM_ gene clearance in the gut resistome in patients colonized with MDROs.

## Data Availability

The datasets used and/or analysed during the study are available from the corresponding author on reasonable request.

## Abbreviations

MDROs: Multidrug-resistant organisms
VRE: Vancomycin-resistant enterococci
CPE: Carbapenemase-producing Enterobacteriaceae
FMT: Faecal microbiota transplantation
GI: Gastrointestinal
CDI: *Clostridium difficile* Infection
